# Acupuncture as a potential host-directed therapeutic strategy in neuroinfectious diseases: a neuroimmune mechanistic perspective

**DOI:** 10.3389/fmed.2026.1861590

**Published:** 2026-06-04

**Authors:** Yun Jin Kim

**Affiliations:** College of Medicine and Health Sciences, United Arab Emirates University, Al Ain, United Arab Emirates

**Keywords:** acupuncture, anti-inflammatory pathway, blood–brain barrier, host-directed therapy, neuroimmune modulation, neuroinfectious diseases, neuroinflammation

## Abstract

Neuroinfectious diseases remain a major global-health challenge, often resulting in substantial morbidity and mortality despite advances in antimicrobial therapies. Growing evidence indicates that host-mediated immune responses, rather than direct pathogen-induced damage alone, play a central role in determining neurological outcomes. This paradigm shift has driven interest in host-directed therapies (HDTs), which aim to modulate immune responses, limit collateral tissue injury, and promote recovery. Acupuncture, a non-pharmacological intervention with emerging evidence of neuroimmune regulatory effects, has been proposed as a potential candidate within this framework. In this perspective, we examine evidence suggesting that acupuncture may modulate inflammatory signaling, microglial activation, blood–brain barrier (BBB) integrity, and oxidative stress. We synthesize current mechanistic insights and map these effects onto key pathological processes in neuroinfectious diseases, noting that existing evidence derives predominantly from non-infectious models and therefore requires cautious interpretation. Although direct evidence in neuroinfectious contexts remains limited, acupuncture represents a biologically plausible adjunctive strategy warranting systematic investigation. We outline critical research priorities and experimental strategies to evaluate its therapeutic potential in this domain.

## 1 Introduction

Neuroinfectious diseases, including viral encephalitis, bacterial meningitis, and emerging conditions such as neuro-COVID, impose a significant and growing burden on global health systems and affected communities through both acute mortality and long-term neurological sequelae caused by neuroinvasive and neurotropic infections (1, 2). The availability of effective antimicrobial therapies and clinical outcomes often remain suboptimal, with many survivors experiencing long-term neurological sequelae including cognitive impairment, epilepsy, and psychiatric disorders (3–5). A key contributor to this paradox is the dual nature of host immune responses: while essential for pathogen clearance, dysregulated immune activation can drive neuroinflammation, neuronal injury, and disruption of the blood–brain barrier (BBB) (6, 7).

This recognition has prompted a shift toward host-directed therapies (HDTs), which aim to recalibrate host responses rather than directly target pathogens. Unlike conventional antimicrobials, HDTs encompass a broad range of strategies, including biologics, small molecules, and non-pharmacological interventions that target host biology rather than the pathogen directly (8–10). In neuroinfectious diseases, current HDTs remain limited and are largely restricted to broad immunosuppressive agents such as corticosteroids (11, 12). Although these interventions can reduce inflammation, they are often nonspecific and may impair protective immunity (13).

Acupuncture, one of the oldest therapeutic practices, is now widely integrated into modern healthcare and increasingly examined through a biomedical lens as a regulator of neuroimmune function (14, 15). Emerging evidence suggests that it modulates inflammatory signaling, autonomic balance, and central nervous system activity, supporting its potential role as a non-pharmacological HDT (16, 17).

The clinical evidence base is still evolving; the most consistent signals are seen in neurological conditions driven by neuroinflammation and immune dysregulation. Evidence from the literature suggests that in stroke, where neuroinflammation, BBB disruption, and oxidative stress parallel key mechanisms of neuroinfectious diseases, adjunctive acupuncture improves functional recovery and reduces disability beyond standard rehabilitation (18). Similar patterns have been reported in long COVID, with improvements in fatigue, cognitive dysfunction, and headache across pooled RCTs, pointing toward neuroimmune modulation as a probable mechanism (19). More recent reports provide moderate-to-high certainty evidence across conditions characterized by neuroinflammatory and autonomic dysregulation (20). These benefits appear most consistent in disorders where disease severity is driven predominantly by host immune responses rather than by pathogen burden itself, an observation of potential relevance to neuroinfectious diseases, where host-mediated immunopathology similarly contributes to poor outcomes.

Acupuncture is increasingly understood to act through defined neuroimmune pathways rather than nonspecific effects. Peripheral needle stimulation is hypothesized to activate somatosensory afferents that engage central autonomic circuits (21), including vagus nerve–mediated cholinergic anti-inflammatory signaling, leading to downstream modulation of cytokine networks, microglial activation, and BBB integrity (22–24). These effects closely align with the processes that drive neuroinflammatory injury in neuroinfectious diseases, supporting the possibility that acupuncture may be investigated as a supportive and mechanistically plausible HDT. Although direct evidence in neuroinfectious diseases remains limited, several infectious or infection-associated neurological conditions share mechanistic features with the models in which acupuncture has shown immunomodulatory effects. For example, sepsis-associated encephalopathy and neuro-COVID involve microglial activation, cytokine amplification, oxidative stress, and BBB dysfunction, all of which have been identified as potential targets of acupuncture-mediated neuroimmune regulation. These mechanisms provide a biologically plausible rationale for further investigation in neuroinfectious disease contexts.

In this Perspective, we examine the mechanistic possibility for acupuncture as an adjunctive strategy in neuroinfectious diseases and outline a framework for its systematic investigation.

## 2 Host-directed therapy in neuroinfectious diseases

Host-directed therapy is any product that can augment host defense mechanisms or modulate excessive inflammation, or both, leading to improved clinical treatment outcomes as shown by reduced morbidity, mortality, and end-organ damage, and long-term functional recovery (25). It represents a paradigm shift from pathogen-centric approaches toward targeted modulation of host biology (26). Rather than directly eliminating the causative organism, HDTs act on host mechanisms to achieve four broad therapeutic goals: interfering with host factors required by a pathogen for productive replication or persistence; enhancing immune responses that mediate host defense; targeting pathways perturbed by a pathogen that drive hyper-inflammation; and modulating dysregulated responses at the site of pathology (27). This approach is particularly relevant in neuroinfectious diseases, where excessive or dysregulated immune responses contribute substantially to disease severity, often independently of ongoing pathogen burden (28).

Key mechanisms of host-mediated pathology in CNS infections include hyperinflammatory cytokine responses, microglial overactivation, oxidative stress, mitochondrial dysfunction, and disruption of BBB integrity (29). These processes represent critical nodes in host–pathogen interactions within the CNS, where immune responses intended to control infection may paradoxically amplify tissue injury (30). Existing HDTs in neuroinfectious diseases offer proof-of-concept that immune modulation can improve outcomes, while simultaneously exposing the limitations of current approaches. Adjunctive dexamethasone in bacterial meningitis reduces the intrathecal inflammatory cascade triggered by bacterial lysis and has been associated with reduced hearing loss and improved neurological outcomes in pneumococcal meningitis in high-income settings (31, 32). In HIV-associated cryptococcal meningitis, adjunctive interferon-gamma (IFN-γ) immunotherapy significantly accelerated fungicidal clearance from the CSF by enhancing Th1-mediated host defense, demonstrating that HDTs can augment rather than suppress protective immunity (33). Recently, IL-6 receptor blockade has been evaluated in COVID-19-associated hyperinflammatory encephalopathy, reflecting the growing application of precision immunotherapy to CNS infectious complications (34). These studies collectively suggest that targeting the host immune response, rather than the pathogen alone, is a legitimate therapeutic strategy in neuroinfectious diseases.

However, these interventions illustrate a fundamental dilemma: broad immunosuppression risks impairing pathogen clearance, while immune augmentation may exacerbate inflammatory injury. Dexamethasone, for example, aims to reduce inflammation but may also suppress beneficial immune functions; accordingly, its clinical benefit has been inconsistent across patient populations and pathogen types (32). There is a pressing need for strategies that can precisely fine-tune immune responses by attenuating harmful inflammatory pathways while preserving or enhancing host defense mechanisms.

Non-pharmacological HDTs remain largely unexplored in this context, despite their potential advantages in safety, broad systemic regulatory capacity, and accessibility in resource-limited settings. Acupuncture, with its emerging mechanistic evidence of multimodal neuroimmune modulation, may represent a promising candidate within this underexplored therapeutic category (35). These effects appear to be bidirectional and state-dependent rather than uniformly suppressive; acupuncture may modulate host immune responses in a context-sensitive manner, downregulating excessive inflammation while preserving or enhancing baseline immune competence (36). This profile directly addresses the precision gap identified in current HDT approaches. Studies of sepsis-associated encephalopathy (SAE) provide a clinically relevant example in which systemic infection induces secondary CNS neuroinflammation, microglial activation, and BBB disruption (37). Experimental findings suggesting that electroacupuncture may attenuate these responses in SAE models (38) provide indirect and exploratory, though mechanistically informative, support for its potential relevance to neuroinfectious diseases.

Preclinical studies suggest that acupuncture may have adjunctive immunomodulatory effects in sepsis – a condition characterized by a dysregulated host response to infection that parallels key immunopathological features of neuroinfectious diseases. Electroacupuncture at ST36 has been shown to improve survival, attenuate cytokine-mediated organ injury, and restore immune homeostasis through vagal-adrenal and sympathetic-splenic reflex pathways (39). Another review corroborated these findings, reporting that acupuncture alleviates excessive inflammatory responses and reduces organ damage in sepsis via these neuroimmune mechanisms (40). In addition, a meta-analysis of clinical trials supported the efficacy and safety of acupuncture as a complementary therapy for sepsis, reporting improvements in inflammatory markers and organ function (41). The evidence supports the biological plausibility of acupuncture as a non-pharmacological HDT candidate worthy of systematic evaluation in neuroinfectious disease contexts.

## 3 Neuroimmune pathophysiology of brain infections

A mechanistic understanding of neuroimmune processes is essential to contextualize the potential role of acupuncture in neuroinfectious diseases. This section describes each key pathological process and maps it explicitly onto the available acupuncture mechanistic evidence. This dual framing is intentional: the pathological processes described here are not merely background context, but active therapeutic targets for which acupuncture has documented preclinical and, in some cases, clinical activity. The convergence of acupuncture-relevant mechanisms across all four axes of CNS infection-related immunopathology forms the scientific foundation for the host-directed hypothesis advanced in this perspective.

### 3.1 Microglial activation and TLR4/NF-κB/NLRP3 signaling

Microglia are the primary immune effector cells of the CNS, functioning as resident sentinels that continuously survey the neural environment for pathological stimuli (42). In the context of CNS infection, pathogen-associated molecular patterns (PAMPs), such as lipopolysaccharide (LPS) from gram-negative bacteria and viral glycoproteins and damage-associated molecular patterns (DAMPs) bind pattern recognition receptors on microglial surfaces, most prominently Toll-like receptor 4 (TLR4) (43). TLR4 activation initiates downstream signaling through myeloid differentiation primary response 88 (MyD88) and Toll-interleukin 1 receptor domain (TIR)-containing adaptor inducing IFN-β (TRIF) adaptor proteins, driving NF-κB nuclear translocation and the transcription of pro-inflammatory cytokines including IL-1β, TNF-α, and IL-6 (44). NLRP3 inflammasome assembly triggered by danger signals such as extracellular ATP and reactive oxygen species produces caspase-1-mediated cleavage of pro-IL-1β into its active form, further amplifying the neuroinflammatory cascade (45). While this initial microglial response is essential for pathogen clearance, sustained or dysregulated activation drives neuronal damage, synaptic dysfunction, and chronic neuroinflammation that persists beyond the infectious episode. Importantly, microglial responses are now understood to reflect a highly heterogeneous functional continuum rather than discrete M1/M2 polarization status—single-cell transcriptomic studies have revealed context-dependent, region-specific, and temporally dynamic activation profiles that should be considered in both disease modeling and therapeutic targeting (46).

Electroacupuncture (EA) engages this pathological axis at multiple mechanistic levels, establishing microglial TLR4/NF-κB/NLRP3 signaling as one of the better characterized candidate targets for acupuncture-mediated modulation in CNS neuroinflammation, at least in preclinical settings. EA at the ST36 (Zusanli) acupoint has been shown to downregulate TLR4 expression on microglial surfaces, reduce MyD88 and TRAF6 protein levels, and suppress NF-κB nuclear translocation, resulting in significant attenuation of IL-1β, TNF-α, and IL-6 production in multiple neuroinflammatory models (47, 48). Furthermore, EA has been shown to inhibit the TLR4/TRIF/MyD88 pathway to modulate microglial polarization, promoting functional shifts toward anti-inflammatory and reparative states characterized by elevated IL-10 and IL-4 and reduced M1-associated markers as demonstrated in traumatic brain injury models (49). The NLRP3 inflammasome represents an additional downstream acupuncture target: EA has been shown to inhibit NLRP3/ASC/caspase-1 assembly via multiple mechanisms, including cannabinoid CB2 receptor-mediated autophagy enhancement in macrophages and microglia, reducing IL-1β and IL-18 maturation and attenuating pyroptotic cell death (50). In the context of postoperative neuroinflammation, a model of sterile CNS immune activation that shares mechanistic features with infection-related neuroinflammation, EA treatment at GV20 significantly reduced hippocampal NLRP3 expression, IL-1β, IL-6, and NF-κB activation, and improved cognitive outcomes (51). These findings across multiple CNS injury paradigms provide preclinical evidence supporting the potential relevance of EA-mediated modulation of the TLR4/NF-κB/NLRP3 axis in neuroinfectious disease-related immunopathology.

### 3.2 Cytokine-mediated neuroinflammation

Amplified cytokine signaling within the CNS and periphery, driven by dysregulated activation of the NLRP3 inflammasome and downstream IL-1β/IL-18 release, represents a central mechanism of immunopathology in neuroinfectious diseases (52). This hyperinflammatory response observed in viral encephalitis, bacterial meningitis, sepsis-associated encephalopathy (SAE), and neuro-COVID involves a pathological cascade in which PAMPs and DAMPs activate TLR4 and NLR family receptors, triggering NF-κB and MAPK signaling pathways that drive the release of TNF-α, IL-1β, IL-6, and IL-17 (53, 54). The NLRP3 inflammasome, formed by the binding of NLRP3 to the ASC adaptor and pro-caspase-1, represents a critical intracellular amplification node: its activation produces caspase-1-driven maturation of IL-1β and IL-18, as well as gasdermin D-mediated pyroptotic cell death, which further releases intracellular DAMPs to perpetuate the inflammatory cycle (55). This self-reinforcing cascade, when uncontrolled, is directly responsible for the neurological deterioration and long-term cognitive sequelae observed in survivors of neuroinfectious diseases. The term ‘cytokine storm,’ while imprecise, captures the clinical manifestation of this process when systemic and intrathecal cytokine amplification overwhelms regulatory mechanisms (56).

Acupuncture’s ability to modulate components of this cytokine amplification cascade may be relevant to its evaluation as a candidate host-directed therapy. EA has been shown to suppress NLRP3 inflammasome activation via multiple independent pathways: through CB2 receptor-mediated autophagy induction in macrophages and microglia, through inhibition of NF-κB/MAPK signaling upstream of NLRP3 assembly, and through upregulation of the cholinergic anti-inflammatory pathway via α7 nicotinic acetylcholine receptors (α7nAChR), which directly suppress the production of TNF-α, IL-1β, and IL-6 in activated immune cells (57, 58). EA has been investigated directly in SAE, the neurological complication of systemic infection mechanistically related to neuroinfectious diseases with multiple lines of evidence supporting its efficacy. In LPS-induced SAE rat models, EA at GV20 and ST36 attenuated microglial activation in hippocampal dentate gyrus and CA1 regions, reduced HMGB1/TLR4/NF-κB-driven neuroinflammation, and improved working memory performance (59). EA also protected against cognitive dysfunction in CLP-induced sepsis models by reducing hippocampal acetylcholinesterase activity, increasing acetylcholine and α7nAChR expression, and attenuating oxidative stress and neuroinflammation in the hippocampus (60). These findings raise the possibility that acupuncture may act not merely as a general anti-inflammatory agent, but as a modulator of the specific components of the NLRP3-driven cytokine cascade that are implicated in neuroinfectious disease immunopathology—a distinction that warrants further investigation in infection-specific models.

### 3.3 Blood–brain barrier disruption

The blood–brain barrier (BBB) is a critical regulator of CNS homeostasis, composed of specialized brain microvascular endothelial cells connected by tight junction (TJ) protein complexes including claudin-5, occludin, and ZO-1—supported by pericytes, astrocytic endfeet, and the basement membrane (61). During CNS infection, pro-inflammatory mediators, particularly HMGB1, TNF-α, IL-1β, and IL-6 disrupt TJ protein expression through multiple converging mechanisms: activation of the HMGB1/TLR4/NF-κB axis drives direct transcriptional downregulation of claudin-5 and occludin; upregulation of matrix metalloproteinase-9 (MMP-9) enzymatically degrades TJ proteins and extracellular matrix components; and RAGE/NADPH oxidase-mediated oxidative stress further destabilizes endothelial junctions (62, 63). The resulting increase in paracellular permeability facilitates the infiltration of peripheral leukocytes, immunoglobulins, and systemic inflammatory mediators into the CNS parenchyma, creating a self-amplifying neuroinflammatory cycle. BBB disruption is a defining feature of bacterial meningitis, viral encephalitis, and tuberculous meningitis, and its severity correlates strongly with neurological outcome and the risk of long-term sequelae (64).

Acupuncture has emerging preclinical evidence for BBB-protective effects that are mechanistically aligned with these pathological processes. A preclinical meta-analysis demonstrated that acupuncture significantly reduced BBB permeability across multiple brain injury models, with concurrent upregulation of occludin, claudin-5, and ZO-1; inhibition of MMP-9 activity; and reduction of glial activation markers (Iba-1, GFAP) and inflammatory mediators (IL-1β, IL-6, TNF-α) (65). EA at GV20 and ST36 has been shown to preserve BBB integrity by inhibiting the HMGB1/TLR4 and RAGE/NADPH oxidase signaling pathways—the same pathways implicated in infection-induced BBB disruption—and by suppressing downstream NF-κB-mediated downregulation of TJ proteins (66). In an SAE model specifically, EA at GV20 and ST36 significantly attenuated LPS-induced HMGB1 release in the hippocampus, reduced RAGE and TLR4 protein expression at the BBB, and preserved TJ complex integrity alongside improvements in cognitive function (67). EA has also been shown to reduce MMP-9-mediated matrix degradation and regulate aquaporin-4 expression to attenuate cerebral edema associated with neuroinflammation (68). These effects on the HMGB1/TLR4/NF-κB axis, which is activated during both infectious and sterile CNS inflammation, provide a mechanistically plausible rationale for evaluating acupuncture as a BBB-stabilizing adjunct in neuroinfectious diseases, where barrier disruption amplifies the severity of host-mediated CNS injury.

### 3.4 Oxidative stress and mitochondrial dysfunction

Reactive oxygen species (ROS) generated during microglial and neutrophil activation represent a critical and frequently underappreciated component of host-mediated neuronal injury in CNS infections (69). During infection, the oxidative burst of activated immune cells produces superoxide anion, hydrogen peroxide, and hydroxyl radicals that overwhelm neuronal antioxidant defenses, initiating a cascade of lipid peroxidation, protein carbonylation, and mitochondrial membrane damage (70). Depletion of glutathione (GSH) and inactivation of glutathione peroxidase 4 (GPX4), the primary phospholipid peroxide repair enzyme, allows accumulation of toxic lipid peroxides, triggering ferroptosis: a form of iron-dependent, regulated oxidative cell death characterized by mitochondrial shrinkage, membrane rupture, and cristae loss (52). The interaction between ROS-mediated oxidative injury and neuroinflammation is bidirectional and self-amplifying: oxidative stress activates the NLRP3 inflammasome and NF-κB signaling, driving further cytokine production; conversely, pro-inflammatory cytokines particularly TNF-α and IL-1β enhance mitochondrial ROS generation through mitochondrial complex I inhibition and upregulation of NADPH oxidase activity (71).

Acupuncture has been shown in preclinical models to engage the Nrf2/HO-1/GPX4 antioxidant axis, making oxidative stress and ferroptosis candidate targets with emerging mechanistic support for acupuncture-based host-directed therapy. The Nrf2 transcription factor, a master regulator of cellular antioxidant defense, controls expression of HO-1, SOD, catalase, GPX4, and ferritin, collectively restoring redox homeostasis under inflammatory stress. EA has been consistently shown to activate Nrf2 nuclear translocation, upregulate HO-1 and SIRT1 expression, increase GPX4 and glutathione levels, and reduce markers of lipid peroxidation including malondialdehyde (MDA) and ROS in multiple CNS neuroinflammatory contexts (72). In ischemia–reperfusion models, which share the oxidative and neuroinflammatory pathophysiology of CNS infection, EA at GV20, ST36, and PC6 activated the Nrf2/SLC7A11/GPX4 axis to inhibit ferroptosis, preserved mitochondrial morphology, reduced cerebral infarct volume, and improved neurological outcomes (73). EA also reduced NLRP3/ASC/caspase-1-mediated neuronal pyroptosis, a complementary form of oxidative inflammatory cell death through mitochondrial protective effects (74). These effects on the Nrf2 antioxidant pathway are particularly relevant to neuroinfectious diseases because the hyperinflammatory cytokine responses characteristic of severe CNS infection directly deplete Nrf2 and GSH through oxidative consumption, creating a state of antioxidant deficiency that acupuncture may help to redress without requiring broad immunosuppression (75).

The pathological processes described in this section, including microglial TLR4/NF-κB activation, NLRP3-driven cytokine amplification, BBB tight junction disruption, and Nrf2/GPX4 antioxidant depletion, form an interconnected, self-reinforcing cycle of neuroinflammation and tissue injury that determines morbidity and mortality in neuroinfectious diseases (76, 77). A potentially important feature of acupuncture in this context is its reported modulatory activity across several nodes of this cycle: suppressing TLR4/NF-κB/NLRP3 signaling, attenuating HMGB1-driven BBB disruption, and activating Nrf2-mediated antioxidant and mitochondrial protection (67). This convergence across multiple mechanistic axes may be advantageous for a host-directed adjunctive strategy in neuroinfectious diseases, where the multiplicity and interdependence of immunopathological mechanisms have limited single-target pharmacological approaches.

## 4 Limitations and scientific gaps

Despite its mechanistic plausibility, several important limitations need to be considered. First, direct evidence supporting the application of acupuncture in neuroinfectious diseases remains limited, as most available studies have been conducted in non-infectious or chronic neurological models, thereby limiting disease-specific translation. Second, substantial heterogeneity in acupuncture protocols, including acupoint selection, stimulation parameters, frequency, and treatment duration, poses significant challenges for reproducibility and cross-study comparability. Third, the interaction between acupuncture-induced immunomodulation and pathogen clearance remains poorly understood. While attenuation of excessive inflammation may be beneficial, there is a potential risk that inappropriate immune suppression could impair antimicrobial defense, particularly in the acute phase of infection. Defining the therapeutic window and immunological thresholds for safe intervention therefore represents a critical unmet need. Current evidence is largely derived from reductionist experimental systems that do not fully capture the complexity of host–pathogen interactions within the CNS and have not yet been integrated with advanced approaches, including single-cell and spatial transcriptomics that would be necessary to resolve cell-type-specific and context-dependent neuroimmune responses. Furthermore, standardized infection models incorporating clinically relevant pathogens are scarce, limiting the translational relevance of preclinical findings. Finally, many existing studies are constrained by small sample sizes, insufficient controls, and variability in experimental design. Rigorous, well-controlled, and standardized preclinical and clinical studies are therefore essential to establish reproducibility, delineate underlying mechanisms with greater specificity, and rigorously evaluate the safety and efficacy of acupuncture as a host-directed therapeutic strategy. Current evidence thus remains preliminary and largely extrapolated from non-infectious neuroinflammatory models; although these models share overlapping neuroimmune pathways with neuroinfectious diseases, their translational applicability remains uncertain and should be interpreted cautiously.

## 5 Future directions

Preclinical studies should prioritize infection-specific models that recapitulate clinically relevant host–pathogen interactions, including both acute and chronic neuroinfectious states. These models should enable detailed characterization of microglial dynamics, BBB integrity, and neuroimmune signaling in response to acupuncture interventions. Incorporation of pathogen-specific variables and disease stage–dependent analyses will be critical to define context-specific effects.

Clinical translation will require well-designed randomized controlled trials evaluating acupuncture as an adjunctive therapy. Such studies should incorporate biomarker-driven endpoints, including cytokine profiles, neurodegeneration markers, and measures of BBB permeability, alongside standardized and reproducible treatment protocols. Stratification by disease severity and timing of intervention may further clarify therapeutic windows and optimize clinical outcomes.

Systems biology approaches will play a central role in bridging mechanistic insights and translational applications. Integration of multi-omics platforms, including single-cell transcriptomics, spatial profiling, and immunometabolism with advanced neuroimaging will enable the identification of molecular signatures and network-level changes associated with acupuncture response.

Finally, future research should aim to define the neural circuits and signaling pathways underlying acupuncture-mediated immunomodulation. These pathways will facilitate the development of precision-based acupuncture strategies, enabling targeted and reproducible interventions tailored to specific neuroinfectious contexts. Such integrative efforts will be essential to transition acupuncture from a mechanistically supported concept to a rigorously validated host-directed therapeutic strategy.

## 6 Conclusion

Acupuncture may be understood as a form of peripheral neuromodulation capable of influencing neuroimmune responses through defined somatosensory and autonomic circuits—mechanisms of potential relevance to the immunopathology of neuroinfectious diseases. By engaging neuroimmune circuits, attenuating excessive inflammation, and preserving BBB integrity, it may influence key mechanisms of host-mediated pathology that are not fully addressed by conventional antimicrobial therapies. Positioning acupuncture within the framework of host-directed therapy shifts its role beyond a traditional intervention toward a mechanistically grounded strategy for modulating host–pathogen interactions in the central nervous system. In this context, it may act as a systems-level regulator of neuroimmune homeostasis, offering a potential complementary approach to support host resilience while limiting immunopathology. However, translating this concept into clinical practice remains challenging. Rigorous validation is needed, including standardized intervention protocols and biomarker-driven clinical studies to define efficacy, safety, and optimal therapeutic windows. Integrating acupuncture within a systems biology and neuroimmune framework could help clarify its mechanisms of action and inform the design of precision-based host-directed interventions. More broadly, this may highlight the potential contribution of neuromodulatory strategies to infectious disease therapeutics. Acupuncture should therefore be viewed not as an established therapy for neuroinfectious diseases, but as a mechanistically plausible adjunctive strategy requiring rigorous infection-specific validation.

## Data Availability

The original contributions presented in the study are included in the article/supplementary material, further inquiries can be directed to the corresponding author.

## References

[1] Misra UK. Specialty grand challenge in neuroinfectious diseases. Front Neurol. (2025) 16:1557610. doi: 10.3389/fneur.2025.1557610, 40027168 PMC11867942

[2] Roberts JA Kapadia RK Pastula DM Thakur KT. Public health trends in neurologically relevant infections: a global perspective. Ther Adv Infect Dis. (2024) 11:20499361241274206. doi: 10.1177/20499361241274206, 39301451 PMC11412215

[3] Kwon CS Rafati A Ottman R Christensen J Kanner AM Jetté N . Psychiatric comorbidities in persons with epilepsy compared with persons without epilepsy: a systematic review and meta-analysis. JAMA Neurol. (2025) 82:72–84. doi: 10.1001/jamaneurol.2024.3976, 39585664 PMC11589854

[4] Yolken R. Infections and neuropsychiatric disorders: new studies document pathways to prevention and treatment. Mol Psychiatry. (2023) 28:2624–6. doi: 10.1038/s41380-023-02072-5, 37106118 PMC10134699

[5] Zareifopoulos N Panayiotakopoulos G. Neuropsychiatric effects of antimicrobial agents. Clin Drug Investig. (2017) 37:423–37. doi: 10.1007/s40261-017-0498-z, 28197902

[6] Müller L Di Benedetto S Müller V. The dual nature of neuroinflammation in networked brain. Front Immunol. (2025) 16:1659947. doi: 10.3389/fimmu.2025.1659947, 40909282 PMC12404926

[7] Zhao Q Chen BA Ai WQ Brazhe N Liu D Zhu LQ . The immunological paradox of CD4^+^ T cells in traumatic brain injury. Int J Surg. (2026) 112:4366–86. doi: 10.1097/JS9.0000000000003689, 41092449

[8] Goletti D Ong CWM Friedland JS. Host-directed therapies: old and new approaches for the treatment of infections. Int J Infect Dis. (2024) 146:107130. doi: 10.1016/j.ijid.2024.107130, 38857650

[9] Kaufmann SHE Dorhoi A Hotchkiss RS Bartenschlager R. Host-directed therapies for bacterial and viral infections. Nat Rev Drug Discov. (2018) 17:35–56. doi: 10.1038/nrd.2017.162, 28935918 PMC7097079

[10] Thom RE D'Elia RV. Future applications of host direct therapies for infectious disease treatment. Front Immunol. (2024) 15:1436557. doi: 10.3389/fimmu.2024.1436557, 39411713 PMC11473292

[11] Gholap AD Khuspe PR Pardeshi SR Uddin MJ Das U Hatvate NT . Achieving optimal health with host-directed therapies (HDTs) in infectious diseases—a new horizon. Adv Ther. (2025) 8:2400169. doi: 10.1002/adtp.202400169

[12] Shin DH. Corticosteroid treatment for central nervous system infections. J Neurocrit Care. (2017) 10:69–75. doi: 10.18700/jnc.170024

[13] Nash TE Ware JM Coyle CM Mahanty S. Etanercept to control inflammation in the treatment of complicated Neurocysticercosis. Am J Trop Med Hyg. (2019) 100:609–16. doi: 10.4269/ajtmh.18-0795, 30608049 PMC6402894

[14] Zhang J Li Z Li Z Li J Hu Q Xu J . Progress of acupuncture therapy in diseases based on magnetic resonance image studies: a literature review. Front Hum Neurosci. (2021) 15:694919. doi: 10.3389/fnhum.2021.694919, 34489662 PMC8417610

[15] Zhang L Luo Y Wei T Wang D He Z Lan L . Acupuncture's multisystem Neuroimmunomodulation: central-peripheral interactions in Gastroenteric, psychiatric, and chronic pain disorders. CNS Neurosci Ther. (2025) 31:e70625. doi: 10.1111/cns.70625, 41199561 PMC12592691

[16] Li N Guo Y Gong Y Zhang Y Fan W Yao K . The anti-inflammatory actions and mechanisms of acupuncture from Acupoint to target organs via neuro-immune regulation. J Inflamm Res. (2021) 14:7191–224. doi: 10.2147/JIR.S341581, 34992414 PMC8710088

[17] Yang L Zhou D Cao J Shi F Zeng J Zhang S . Revealing the biological mechanism of acupuncture in alleviating excessive inflammatory responses and organ damage in sepsis: a systematic review. Front Immunol. (2023) 14:1242640. doi: 10.3389/fimmu.2023.1242640, 37753078 PMC10518388

[18] Zhong X Luo X Li L Liu J Sun X Zhang H. Effects of acupuncture on ischemic stroke: a systematic review with meta-analyses and trial sequential analyses. Complement Ther Clin Pract. (2024) 57:101905. doi: 10.1016/j.ctcp.2024.101905, 39276664

[19] Lam WC Wei D Li H Yao L Zhang S Lai MXY . The use of acupuncture for addressing neurological and neuropsychiatric symptoms in patients with long COVID: a systematic review and meta-analysis. Front Neurol. (2024) 15:1406475. doi: 10.3389/fneur.2024.1406475, 39099786 PMC11294104

[20] Dou B Li Y Ma J Xu Z Fan W Tian L . Role of Neuroimmune crosstalk in mediating the anti-inflammatory and analgesic effects of acupuncture on inflammatory pain. Front Neurosci. (2021) 15:695670. doi: 10.3389/fnins.2021.695670, 34408622 PMC8366064

[21] Valle G. Peripheral neurostimulation for encoding artificial somatosensations. Eur J Neurosci. (2022) 56:5888–901. doi: 10.1111/ejn.15822, 36097134 PMC9826263

[22] Kupari J Ernfors P. Pricking into autonomic reflex pathways by electrical acupuncture. Neuron. (2020) 108:395–7. doi: 10.1016/j.neuron.2020.09.026, 33181070

[23] Liu S Wang Z Su Y Qi L Yang W Fu M . A neuroanatomical basis for electroacupuncture to drive the vagal-adrenal axis. Nature. (2021) 598:641–5. doi: 10.1038/s41586-021-04001-4, 34646018 PMC9178665

[24] Liu FJ Wu J Gong LJ Yang HS Chen H. Non-invasive vagus nerve stimulation in anti-inflammatory therapy: mechanistic insights and future perspectives. Front Neurosci. (2024) 18:1490300. doi: 10.3389/fnins.2024.1490300, 39605787 PMC11599236

[25] Kiran D Podell BK Chambers M Basaraba RJ. Host-directed therapy targeting the *Mycobacterium tuberculosis* granuloma: a review. Semin Immunopathol. (2016) 38:167–83. doi: 10.1007/s00281-015-0537-x, 26510950 PMC4779125

[26] Zumla A Rao M Wallis RS Kaufmann SH Rustomjee R Mwaba P . Host-directed therapies for infectious diseases: current status, recent progress, and future prospects. Lancet Infect Dis. (2016) 16:e47–63. doi: 10.1016/S1473-3099(16)00078-5 27036359 PMC7164794

[27] Cai J Zhou H Liu M Zhang D Lv J Xue H . Host immunity and intracellular bacteria evasion mechanisms: enhancing host-directed therapies with drug delivery systems. Int J Antimicrob Agents. (2025) 65:107492. doi: 10.1016/j.ijantimicag.2025.107492, 40107461

[28] Ardakani R Jia L Matthews E Thakur KT. Therapeutic advances in neuroinfectious diseases. Ther Adv Infect Dis. (2024) 11:20499361241274246. doi: 10.1177/20499361241274246, 39314743 PMC11418331

[29] Huang X Hussain B Chang J. Peripheral inflammation and blood-brain barrier disruption: effects and mechanisms. CNS Neurosci Ther. (2021) 27:36–47. doi: 10.1111/cns.13569, 33381913 PMC7804893

[30] Wallis RS O'Garra A Sher A Wack A. Host-directed immunotherapy of viral and bacterial infections: past, present and future. Nat Rev Immunol. (2023) 23:121–33. doi: 10.1038/s41577-022-00734-z, 35672482 PMC9171745

[31] de Gans J van de Beek D European Dexamethasone in Adulthood Bacterial Meningitis Study Investigators. Dexamethasone in adults with bacterial meningitis. N Engl J Med. (2002) 347:1549–56. doi: 10.1056/NEJMoa021334, 12432041

[32] van de Beek D Farrar JJ de Gans J Mai NT Molyneux EM Peltola H . Adjunctive dexamethasone in bacterial meningitis: a meta-analysis of individual patient data. Lancet Neurol. (2010) 9:254–63. doi: 10.1016/S1474-4422(10)70023-5, 20138011 PMC2835871

[33] Jarvis JN Meintjes G Rebe K Williams GN Bicanic T Williams A . Adjunctive interferon-γ immunotherapy for the treatment of HIV-associated cryptococcal meningitis: a randomized controlled trial. AIDS (London, England). (2012) 26:1105–13. doi: 10.1097/QAD.0b013e3283536a93, 22421244 PMC3640254

[34] Paterson RW Brown RL Benjamin L Nortley R Wiethoff S Bharucha T . The emerging spectrum of COVID-19 neurology: clinical, radiological and laboratory findings. Brain J Neurol. (2020) 143:3104–20. doi: 10.1093/brain/awaa240, 32637987 PMC7454352

[35] Wang M Liu W Ge J Liu S. The immunomodulatory mechanisms for acupuncture practice. Front Immunol. (2023) 14:1147718. doi: 10.3389/fimmu.2023.1147718, 37090714 PMC10117649

[36] Yu WL Park JY Park HJ Kim SN. Changes of local microenvironment and systemic immunity after acupuncture stimulation during inflammation: a literature review of animal studies. Front Neurol. (2023) 13:1086195. doi: 10.3389/fneur.2022.1086195, 36712435 PMC9875056

[37] Zheng SM Zhao FL Luo YY Lin XF Wen MY. Clinical effect of electroacupuncture at Baihui and Shuigou points in treatment of brain injury in patients with sepsis-associated encephalopathy. Acupunct Res. (2020) 45:402–6. doi: 10.13702/j.1000-0607.190781, 32447856

[38] Li C Yu TY Zhang Y Wei LP Dong SA Shi J . Electroacupuncture improves cognition in rats with sepsis-associated encephalopathy. J Surg Res. (2020) 256:258–66. doi: 10.1016/j.jss.2020.06.05 32712439

[39] Pan WX Fan AY Chen S Alemi SF. Acupuncture modulates immunity in sepsis: toward a science-based protocol. Auton Neurosci. (2021) 232:102793. doi: 10.1016/j.autneu.2021.102793, 33684727

[40] Li XY Li CQ Wang JW Ma JN Kuang XB Zhang JY . Acupuncture as an adjunctive therapy for sepsis: advantages, limitations, and strategie. World J Acupunct-Moxibustion. (2026) 36:51–60. doi: 10.1016/j.wjam.2025.10.001

[41] Xian J Wang L Zhang C Wang J Zhu Y Yu H . Efficacy and safety of acupuncture as a complementary therapy for sepsis: a systematic review and meta-analysis. Acupunct Med. (2023) 41:3–15. doi: 10.1177/09645284221086288, 35579024

[42] Gao C Jiang J Tan Y Chen S. Microglia in neurodegenerative diseases: mechanism and potential therapeutic targets. Signal Transduct Target Ther. (2023) 8:359. doi: 10.1038/s41392-023-01588-0, 37735487 PMC10514343

[43] Chen R Zou J Chen J Zhong X Kang R Tang D. Pattern recognition receptors: function, regulation and therapeutic potential. Signal Transduct Target Ther. (2025) 10:216. doi: 10.1038/s41392-025-02264-1, 40640149 PMC12246121

[44] Pi W Cao L Wang Q Yong VW Yang L Xue M. The TLR4/MyD88 axis: orchestrator of neuroinflammation from molecular initiation to systemic crosstalk. Brain Hemorrhages. (2026). doi: 10.1016/j.hest.2026.03.002

[45] Kelley N Jeltema D Duan Y He Y. The NLRP3 Inflammasome: an overview of mechanisms of activation and regulation. Int J Mol Sci. (2019) 20:3328. doi: 10.3390/ijms20133328, 31284572 PMC6651423

[46] Ramirez AI de Hoz R Salobrar-Garcia E Salazar JJ Rojas B Ajoy D . The role of microglia in retinal neurodegeneration: Alzheimer's disease, Parkinson, and Glaucoma. Front Aging Neurosci. (2017) 9:214. doi: 10.3389/fnagi.2017.00214, 28729832 PMC5498525

[47] Oh JE Kim SN. Anti-inflammatory effects of acupuncture at ST36 point: a literature review in animal studies. Front Immunol. (2022) 12:813748. doi: 10.3389/fimmu.2021.813748, 35095910 PMC8790576

[48] Ye HM Li ZY Zhang P Kang Z Zhou DS. Exploring mechanism of Electroacupuncture in modulating Neuroinflammation based on intestinal Flora and its metabolites. Chin J Integr Med. (2025) 31:183–92. doi: 10.1007/s11655-024-3766-9, 39039343

[49] Cao LX Lin SJ Zhao SS Wang SQ Zeng H Chen WA . Effects of acupuncture on microglial polarization and the TLR4/TRIF/MyD88 pathway in a rat model of traumatic brain injury. Acupunct Med J Br Med Acupunct Soc. (2023) 41:235–45. doi: 10.1177/09645284221108214, 36046956

[50] Tang B Li Y Xu X Du G Wang H. Electroacupuncture ameliorates neuronal injury by NLRP3/ASC/Caspase-1 mediated Pyroptosis in cerebral ischemia-reperfusion. Mol Neurobiol. (2024) 61:2357–66. doi: 10.1007/s12035-023-03712-1, 37874480

[51] Zhao W Zou W. Effects of electroacupuncture on postoperative cognitive dysfunction and its underlying mechanisms: a literature review of rodent studies. Front Aging Neurosci. (2024) 16:1384075. doi: 10.3389/fnagi.2024.1384075, 38596595 PMC11002135

[52] Xu W Huang Y Zhou R. NLRP3 inflammasome in neuroinflammation and central nervous system diseases. Cell Mol Immunol. (2025) 22:341–55. doi: 10.1038/s41423-025-01275-w, 40075143 PMC11955557

[53] Barbosa-Silva MC Lima MN Battaglini D Robba C Pelosi P Rocco PRM . Infectious disease-associated encephalopathies. Crit Care. (2021) 25:236. doi: 10.1186/s13054-021-03659-6, 34229735 PMC8259088

[54] Hong Y Chen P Gao J Lin Y Chen L Shang X. Sepsis-associated encephalopathy: from pathophysiology to clinical management. Int Immunopharmacol. (2023) 124:110800. doi: 10.1016/j.intimp.2023.110800, 37619410

[55] Huang Y Xu W Zhou R. NLRP3 inflammasome activation and cell death. Cell Mol Immunol. (2021) 18:2114–27. doi: 10.1038/s41423-021-00740-6, 34321623 PMC8429580

[56] Karki R Netea MG Diorio C Kanneganti TD. Cytokine storm. Nat Rev Dis Primers. (2026) 12:1. doi: 10.1038/s41572-025-00677-4, 41540062

[57] Su R Wang Z Zhang G Tao Y Zhu H Chen H . The analgesic effect of NLRP3 Inflammasome in the relief of inflammatory pain by Electroacupuncture: a systematic review and Meta-analysis of animal studies. J Pain Res. (2025) 18:4413–30. doi: 10.2147/JPR.S534757, 40901383 PMC12399899

[58] Sun L Yong Y Wei P Wang Y Li H Zhou Y . Electroacupuncture ameliorates postoperative cognitive dysfunction and associated neuroinflammation via NLRP3 signal inhibition in aged mice. CNS Neurosci Ther. (2022) 28:390–400. doi: 10.1111/cns.13784, 34951130 PMC8841296

[59] Mo Y Wang L Ren M Xie W Ye X Zhou B . Electroacupuncture prevents LPS-induced neuroinflammation via upregulation of PICK-TLR4 complexes in the microglia of hippocampus. Brain Res Bull. (2021) 177:295–304. doi: 10.1016/j.brainresbull.2021.10.010, 34673136

[60] Wang W Chen C Wang Q Ma JG Li YS Guan Z . Electroacupuncture pretreatment preserves telomerase reverse transcriptase function and alleviates postoperative cognitive dysfunction by suppressing oxidative stress and neuroinflammation in aged mice. CNS Neurosci Ther. (2024) 30:e14373. doi: 10.1111/cns.14373, 37501354 PMC10848091

[61] Kadry H Noorani B Cucullo L. A blood-brain barrier overview on structure, function, impairment, and biomarkers of integrity. Fluids Barriers CNS. (2020) 17:69. doi: 10.1186/s12987-020-00230-3, 33208141 PMC7672931

[62] García-Domínguez M. Neuroinflammation: mechanisms, dual roles, and therapeutic strategies in neurological disorders. Curr Issues Mol Biol. (2025) 47:417. doi: 10.3390/cimb47060417, 40699816 PMC12191620

[63] Smith JA Das A Ray SK Banik NL. Role of pro-inflammatory cytokines released from microglia in neurodegenerative diseases. Brain Res Bull. (2012) 87:10–20. doi: 10.1016/j.brainresbull.2011.10.004, 22024597 PMC9827422

[64] Archie SR Al Shoyaib A Cucullo L. Blood-brain barrier dysfunction in CNS disorders and putative therapeutic targets: an overview. Pharmaceutics. (2021) 13:1779. doi: 10.3390/pharmaceutics13111779, 34834200 PMC8622070

[65] Zhang K Liang Y Wang Y Yin T Ming K. Acupuncture improves blood-brain barrier integrity through multi-targeted mechanisms: a preclinical meta-analysis. Front Neurol. (2025) 16:1648117. doi: 10.3389/fneur.2025.1648117, 41281566 PMC12636094

[66] Zhang YM Xu H Sun H Chen SH Wang FM. Electroacupuncture treatment improves neurological function associated with regulation of tight junction proteins in rats with cerebral ischemia reperfusion injury. Evid Based Complement Alternat Med. (2014) 2014:989340. doi: 10.1155/2014/989340, 25009574 PMC4070389

[67] Xin Y Wang J Chu T Zhou Y Liu C Xu A. Electroacupuncture alleviates neuroinflammation by inhibiting the HMGB1 signaling pathway in rats with Sepsis-associated encephalopathy. Brain Sci. (2022) 12:1732. doi: 10.3390/brainsci12121732, 36552192 PMC9776077

[68] Yonglin C Ling O Lingling M Bufan WU Rou P Sitong L . Electroacupuncture ameliorates blood-brain barrier disruption after ischemic stroke through histone acetylation regulation at the matrix metalloproteinase 9 and tissue inhibitor of metalloproteinase 2 genes. J Tradit Chin Med. (2024) 44:734–44. doi: 10.19852/j.cnki.jtcm.20240610.004, 39066534 PMC11337257

[69] Fan H Bai Q Yang Y Shi X Du G Yan J . The key roles of reactive oxygen species in microglial inflammatory activation: regulation by endogenous antioxidant system and exogenous sulfur-containing compounds. Eur J Pharmacol. (2023) 956:175966. doi: 10.1016/j.ejphar.2023.175966, 37549725

[70] Zhu W Dong J Han Y. Electroacupuncture downregulating neuronal Ferroptosis in MCAO/R rats by activating Nrf2/SLC7A11/GPX4 Axis. Neurochem Res. (2024) 49:2105–19. doi: 10.1007/s11064-024-04185-x, 38819696 PMC11233380

[71] Zhou K Shi L Wang Y Chen S Zhang J. Recent advances of the NLRP3 Inflammasome in central nervous system disorders. J Immunol Res. (2016) 2016:1–9. doi: 10.1155/2016/9238290, 27652274 PMC5019917

[72] Tong T Hao C Shen J Liu S Yan S Aslam MS . Electroacupuncture ameliorates chronic unpredictable mild stress-induced depression-like behavior and cognitive impairment through suppressing oxidative stress and neuroinflammation in rats. Brain Res Bull. (2024) 206:110838. doi: 10.1016/j.brainresbull.2023.110838, 38123022

[73] Liu Y Wang Q Hou Z Gao Y Li P. Electroacupuncture inhibits Ferroptosis by modulating Iron metabolism and oxidative stress to alleviate cerebral ischemia-reperfusion injury. J. Mol Neurosci. (2025) 75:63. doi: 10.1007/s12031-025-02355-2, 40317390 PMC12049298

[74] Hou Z Liu Y Wang Q Li P. Electroacupuncture attenuates cerebral ischemia-reperfusion injury by inhibiting Ferroptosis via the p53/SLC7A11 pathway. Clin Exp Pharmacol Physiol. (2025) 52:e70036. doi: 10.1111/1440-1681.70036, 40205608 PMC11982422

[75] Li P Huang W Yan YN Cheng W Liu S Huang Y . Acupuncture can play an antidepressant role by regulating the intestinal microbes and neurotransmitters in a rat model of depression. Med. Sci. Monit. (2021) 27:e929027. doi: 10.12659/MSM.929027, 34039946 PMC8168287

[76] Fornari Laurindo L Aparecido Dias J Cressoni Araújo A Torres Pomini K Machado Galhardi C Rucco Penteado Detregiachi C . Immunological dimensions of neuroinflammation and microglial activation: exploring innovative immunomodulatory approaches to mitigate neuroinflammatory progression. Front Immunol. (2024) 14:1305933. doi: 10.3389/fimmu.2023.1305933, 38259497 PMC10800801

[77] Yang J Wise L Fukuchi KI. TLR4 cross-talk with NLRP3 Inflammasome and complement signaling pathways in Alzheimer's disease. Front Immunol. (2020) 11:724. doi: 10.3389/fimmu.2020.00724, 32391019 PMC7190872

